# Supplementary material for: Human butyrylcholinesterase in Cohn fraction IV-4 purified in a single chromatography step on Hupresin
Source: PLoS One. 2023 Jan 13;18(1):e0280380. doi: 10.1371/journal.pone.0280380 (PMC9838835; doi:10.1371/journal.pone.0280380)
Supplement: S1 Raw images — (PDF) [file pone.0280380.s001.pdf]

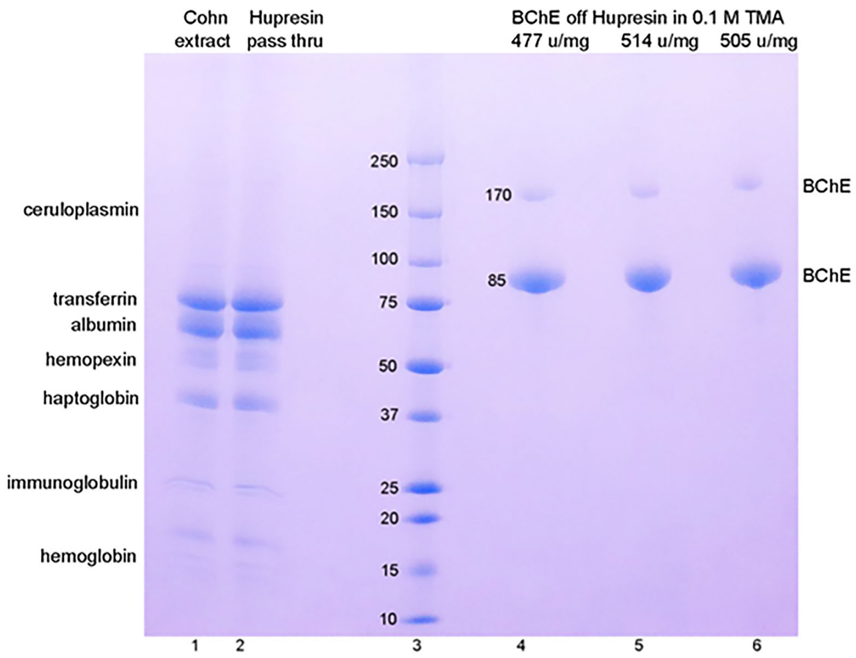

Figure 3 Image was captured with a digital camera. The entire image was used for figure 3.

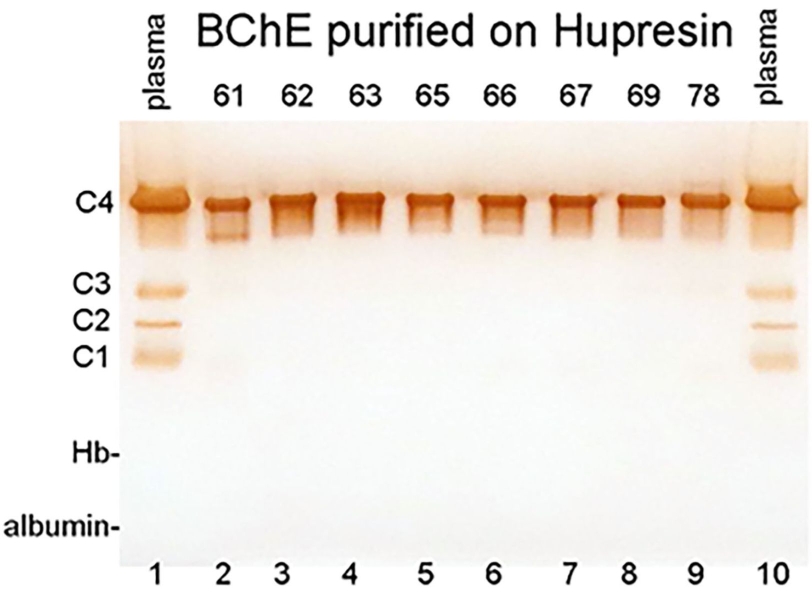

Figure 4. Image was captured with a digital camera. The entire image was used for figure 4.
