# Supplementary material for: Human butyrylcholinesterase in Cohn fraction IV-4 purified in a single chromatography step on Hupresin
Source: PLoS One. 2023 Jan 13;18(1):e0280380. doi: 10.1371/journal.pone.0280380 (PMC9838835; doi:10.1371/journal.pone.0280380)
Supplement: Attachment — Submitted filename: Response to Reviewers comments 11Dec2022.docx [file pone.0280380.s002.docx]

Dr. Bhattarai

Thank you for the reviewer’s comments to our paper “Human butyrylcholinesterase in Cohn fraction IV-4 purified in a single chromatography step on Hupresin”.

Manuscript # PONE-D-22-24553.

We would like to thank the reviewers for their helpful comments.

The original images for gel figures 3 and 4 are included in an S1-RAW-Images file in the Supporting Information. Figures 3 and 4 are the only gel images in the manuscript.

Reviewers comments and responses

Comment 1	Line 517: “contaminant proteins in Chon fraction IV were identified by LC-MS/MS

=> MS-Data for protein identification might be added as Supplementary Information.

Response 1	“LC-MS/MS data were reported in Schopfer et.al [Schopfer, Lockridge, David & Hinrichs, Purification of human butyrylcholinesterase from frozen Cohn fraction IV-4 by ion exchange and Hupresin affinity chromatography, Plos One, doi.org/10.1371/journal pone.0209795]” was added to the first paragraph in section “SDS gel electrophoresis”.

Comment 2	Fig 4: Jugded only from the shown gel it can not be concluded that the protein is a tetramer, since there is no marker used. There are several high molecular weight marker available. Addition of a marker would be very helpful.

Response 2	The pattern of BChE active bands from plasma provide a well establish reference for the oligomer state of BChE, where C1, C2, C3 and C4 reflect monomeric BChE, a disulfide crosslinked dimer of BChE and albumin [Masson Biochem Biophys Acta, 998, 258-266 (1989)], dimer of BChE, and tetrameric BChE. The legend to figure 4 has been amended to emphasize this fact.

Comment 3	The staining of the gel is derived from the activity of BChE. Tetramers , trimers, dimer and monomeric forms were active. However, in lane 2 (sample 62) there is a distinct band clearly visible below the main band, but this band does not correlate to the trimer, dimer or monomer form. This should at least be mentioned and discussed.

Response 3	The extra band in lane 2 is consistent with the BChE trimer. This was mentioned at the end of the last paragraph in the section Tetramer status of BChE purified by chromatography on Hupresin.

Comment 4	Is it possible to stain the gel after the reaction with Coomassie blue?

Response 4	It is possible to stain the gel with Coomassie Blue after the gel has been stained for activity. An example of this is given in figure 9 from Schopfer, Lockridge, David & Hinrichs; Purification of human butyrylcholinesterase from frozen Cohn fraction IV-4 by ion exchange and Hupresin affinity; Plos One doi.org/10.1371/journal.pone.0209795 2019.

Comment 5	Lane 572 PBS should be used for phosphate buffered saline.

Response 5	PBS was substituted for phosphate buffered saline.

Comment 6	It is claimed that the BChE is stable in PBS at 4°C for 20 years. Please give more details on the origin of the samples or Refs. Have the samples all been produced under the same conditions using same reagents?

Response 6	This statement is based on our accumulated experience with BChE produced by various methods. The first sentence of the section “Stability of BChE” has been changed “BChE produced by various methods when stored in PBS, sterile and concentrated (e.g. 6000-7000 u/mL), at 4°C, is stable for up to 20 years [Lockridge Pharmacol Therap 148, 34-46 (2015)].”

Comment 7	Samples shown in Fig 5 are all derived from the new Chon fraction correct ?

Response 7	Correct. We have added a statement to that effect in the legend to figure 5.

Comment 8	Stability assessment was done by activity measurements. Has the integrity of the proteins be checked ? e.g. by MS or analytical SEC?

Response 8	No, we have not checked stability in those ways. We have added the following explanation to the second paragraph in the section “Stability of BChE” to expand on this point. “Stability was assessed by activity assays. Loss of activity might have resulted from oxidation of critical BChE residues or by fragmentation of BChE. It has been reported that fetal bovine acetylcholinesterase can be cleaved at the C-terminus resulting in monomers [Saxena, Hur, Luo & Doctor, Natural monomeric form of fetal bovine serum acetylcholinesterase lacks the C-terminal tetramerization domain; Biochemistry 42, 15292 (2003)]”.

Comment 9	The BChE prepared from the new Chron fraction is less stable than the old batches (up to 20 years at 4°C). This should be mentioned and discussed ? Is there a possible reason for this ?

Response 9	There appears to be some confusion here. Purified BChE is stable for 20 years when stored at 4°C, sterile. BChE in the crude new process Cohn Fraction IV-4 starting material is stable for at least 7 years when stored at -80°C. We have modified figure legend 5 to clarify this confusion. “BChE, purified from new Cohn fraction IV-4, was lyophilized in ….”

Comment 10	Kallikrein activity: There are certain differences between different BChe batches. What is the upper limit for kallikrein kinase activity allowed for human preparations – please add value – as done for LPS level (despite this material is not intended for use in humans).

Response 10	No OSHA Permissible Exposure Limit(PEL) for kallikrein kinase is available, and the safety data sheet information on kallikrein kinase does not list an upper limit for exposure.

Comment 11	Line 671 “… dos not yield pure protein” please give a number.

Response 11	The last paragraph in section “Advantages and disadvantages of affinity chromatography on Hupresin” has been modified to include the statement “It is important to point out that purification of BChE from plasma in a single step with Hupresin yields protein that is only 10-15% pure [Onder, David, Tacal, Schopfer & Lockridge, Hupresin retains binding capacity for butyrylcholinesterase and acetylcholinesterase after sanitation with sodium hydroxide, Front Pharmacol doi: 10.3389/fphar.2017.00713].”

Comment 12	684. The last sentence is a little bit misleading.

Response 12	I don’t see the problem

Comment 13	Is the BChE produced with the new method 99% pure ? Please describe the method for estimating this rate.

Response 13	The following section was added to the Results and Discussion:

**BChE purity**

BChE purified from new process Cohn fraction IV-4 by a single chromatography step on Hupresin affinity resin is 99% pure. Purity was assessed by specific activity using butyrylthiocholine as substrate, where 500 u/mg protein was considered to be 99% pure. Purity was also assessed by contaminating bands seen in SDS PAGE stained with Coomassie blue (Fig 3), where no contaminating bands could be seen.

Comment 14	More analytical data for the purified BChE should be added. E.g. analytical SEC, analytical HIC, MS-Data. HPLC…..

Response 14	MS-data are described in the responses to comments 1 & 13. SEC, HIC and HPLC were not performed.

Comment 15	Maybe it is possible to identify the proline-rich peptide.

Response 15	The following description of the polyproline tetramerization domain was added to the BChE purification” section. “The polyproline peptide (PSPPLPPPPPPPPPPPPPPPPPPPPLP) from RAPH1 (Ras-associated and pleckstrin homology domains-containing protein 1, aka lamellipodin, gi:82581557) that serves to lock-in the BChE tetramer [Li, Schopfer, Masson & Lockridge, Lamellipodin proline rich peptides associated with native plasma butyrylcholinesterase tetramers; Biochem J 411, 425 (2008)] was identified in the mass spectral data.”

Comment 16	As the protein has been washed with Triton x-100: How can you be sure, that Triton X-100 is completely removed and does not stuck to the protein ? I bet it does.

Response 16	Triton X-100 was only used for the solvent/detergent tests. The majority of the purifications were performed without Triton X-100. But we agree that it is very hard to completely remove all of the Triton X-10 after a protein has been exposed. To address this point, we have added the following to last paragraph of section “Solvent/Detergent passes through Hupresin without binding”.  “It is very hard to completely remove all of the Triton X-10 after a protein has been exposed. However, in those instances where Triton X‑100 was used, there is no evidence for practical consequences from residual Triton X-100 on the final purified BChE. For example, Triton X‑100 inhibits BChE [Li, Stribley, Ticu, Xie, Schopfer, Hammond, Brimijoin, Hinrichs & Lockridge, Abundant tissue butyrylcholinesterase and its possible function in the acetylcholinesterase knockout mouse, J Neurochem 75, 1320 (2000)] but the specific activity of the final BChE after purification in the presence or absence of Triton X-100 is the same (see Table 2).”

Minor topics:

Please give sources for “complex” compounds.

Comment 17	Line 176: Source for EDTA

Response 17	“(Sigma-Aldrich cat# E2,629-0, St Louis, MO)” was added

Comment 18	Line 181: Please give conditions for mixing time (rpm, …)

Response 18	“at 60 rpm” was added

Comment 19	Line 276 The abbreviation TMA is used. It should be introduced, when the term is used the first tim (line 76 tetramethylammonium bromide). Please give source.

Response 19	“(TMA, Tokyo Chemical Industry, cat# T0135, Tokyo Japan)” was added to line 76.

Comment 20	Line 211: source for solvents and detergent (tri-(n-butyl) phosphate, Triton X-100)

Response 20	“Tri-(n-butyl) phosphate, 98% was from Alfa Aesar, cat# A16084, 98%, Wardhill, MA and Triton X100 was from Acros Organics/ThermoFisher cat# 9001‑93-1, Pittsburg PA.” was added to line 211.

Comment 21	Line 278: PBS Please explain: is this “self made” PBS (please give composition) or from an supplier (please give source)

Response 21	“PBS (phosphate buffered saline) was prepared in-house. A 10X solution contained 71.2 g Na_2_HPO_4_*2H_2_O, 9.6 g KH_2_PO_4_, 320 g NaCl, and 8 g KCl. After 10-fold dilution this gave 140 mM Na/KCl in 12 mM Na/KPO_4_ pH 7.4.” was added to the last paragraph in the “Buffer exchange” section.

Comment 22	Lines 318/319: Please give source for dithiobisnitrobenzoic acid, and butyrylthiocholine iodide.

Response 22	“(Sigma Cat# D8130, St Louis MO)” was inserted after dithiobisnitrobenzoic acid.

“(Fluka/Honeywell cat# 20820, Charlotte NC)” was inserted after butyrylthiocholine iodide.

Comment 23	Line 325 Source for “bovine albumin” which kind ? fatty acid free ?

Response 23	“(Sigma, bovine fraction V, fatty acid free, cat# A8022, St Louis, MO)” was inserted after bovine albumin.

Comment 24	346 SDS polyacrylamide: Which running buffer was used ? Which marker was used ? please give sources.

Response 24	“Running buffer was 25 mM Tris, 180 mM glycine, and 0.01% sodium dodecyl sulfate. Molecular weight markers were Odyssey one-color protein molecular weight markers from LiCor (Lincoln NE) cat# 928-40000.” was inserted at the end of the paragraph in the “SDS polyacrylamide gel” section.

Comment 25	352: Nondenaturing polyacrylamide gel: Which running buffer was used ?

Response 25	“in 22 mM Tris, 23 mM glycine pH 9” was inserted at the end of the first sentence in the “Nondenaturing polyacrylamide gel stained for BChE activity” section.

Comment 26	428: Citation should be in the reference section. Use number for ref. only

Response 26	citation Holloway, Anal Biochem 53, 304 (1973) has been moved to the reference section.
